# Supplementary material for: Dual Transcriptome Analysis Reveals the Changes in Gene Expression in Both Cotton and Verticillium dahliae During the Infection Process
Source: J Fungi (Basel). 2024 Nov 7;10(11):773. doi: 10.3390/jof10110773 (PMC11595654; doi:10.3390/jof10110773)
Supplement: Supplementary file 1 [file jof-10-00773-s001.zip › jof-3288626-supplementary.pdf]

Figure S1

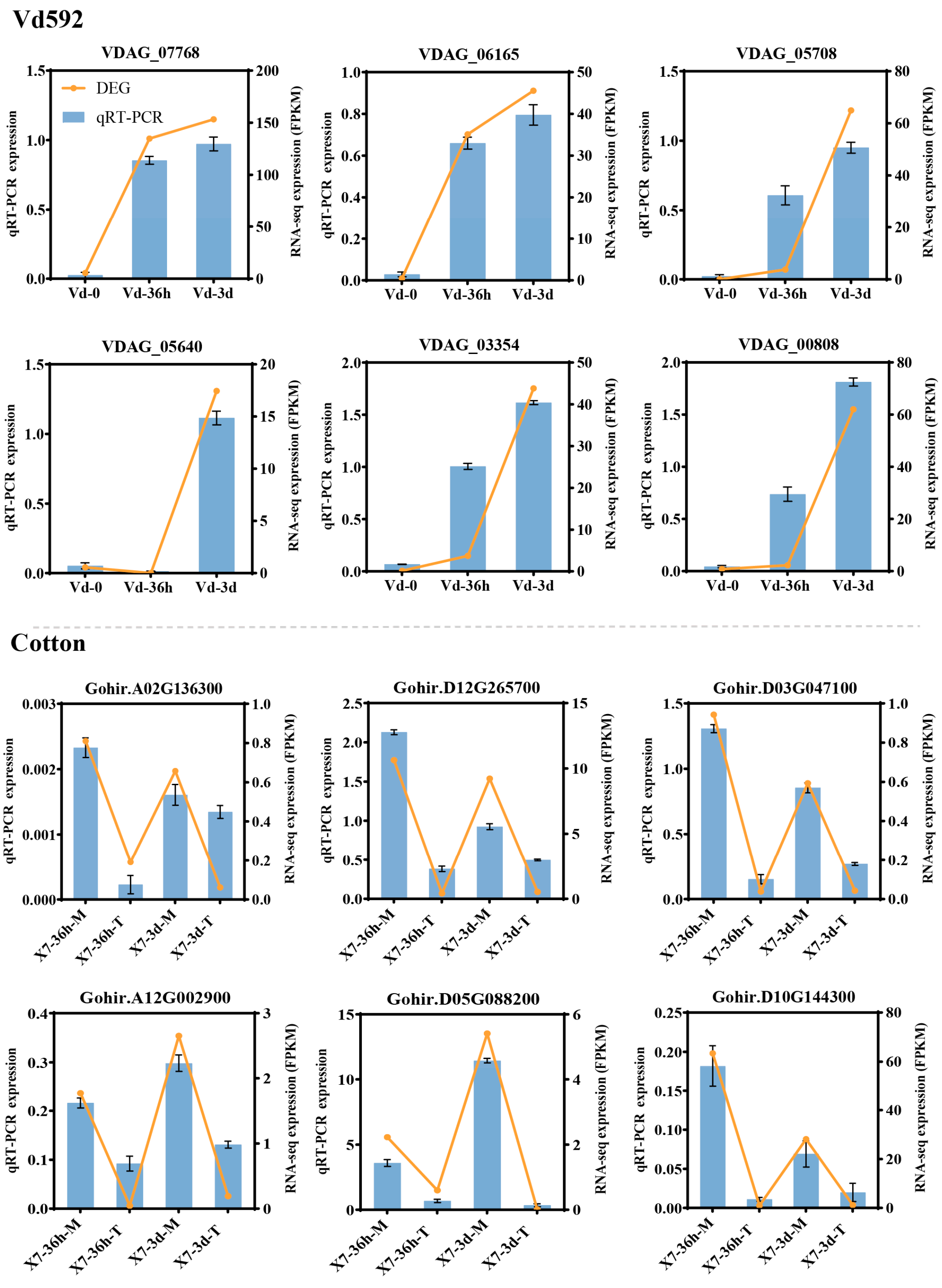

Figure S1. Confirmation of RNA-seq via qRT-PCR analysis. The expression of twelve selected DEGs from the RNA-seg analysis were measured by qRT-PCR in Vd-0, Vd-36h, Vd-3d, X7-36h-M, X7-36h-T, X7-3d-M, and X7-3d-T. The histograms were plotted using data obtained by qRT-PCR and the corresponded line chart was plotted by FPKM values from the RNA-seq analysis. Each bar represents the mean value with standard errors of three independent experiments.

Figure S2

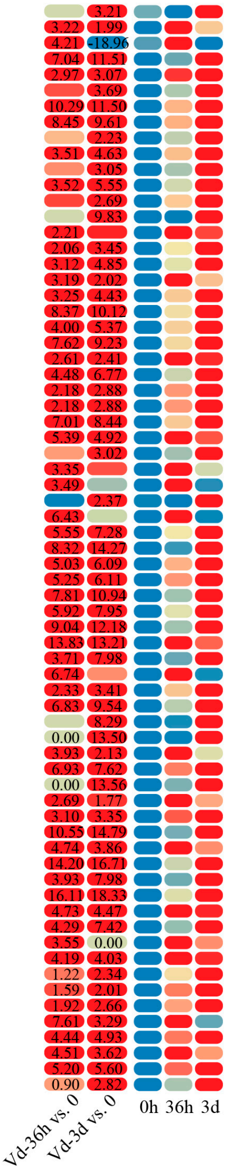

Vd-36h vs. 0	Vd-3d vs. 0	Gene ID	Length	Cys %	Annotation
	3.21	VDAG_06385	186	2.2	AA1-like domain-containing protein
3.22	1.99	VDAG_06675	280	1.4	Auxiliary Activity family 9 catalytic domain-containing protein
4.21	-18.96	VDAG_05096	198	3.5	Lipocalin/cytosolic fatty-acid binding domain-containing protein
7.04	11.51	VDAG_03374	342	2.3	PA14 domain-containing protein
2.97	3.07	VDAG_01759	346	1.2	Pyridoxamine 5'-phosphate oxidase putative domain-containing protein
	3.69	VDAG_01316	390	2.8	Right handed beta helix domain-containing protein
10.29	11.50	VDAG_07135	388	4.1	Starch binding domain-containing protein
8.45	9.61	VDAG_09506	301	2.3	5'/3'-nucleotidase SurE family protein
	2.23	VDAG_00600	351	1.1	Acetyl esterase
3.51	4.63	VDAG_07784	295	5.4	Acetylxylan esterase
	3.05	VDAG_06538	271	1.5	Carbonic anhydrase
3.52	5.55	VDAG_02100	230	1.7	CAS1 protein
	2.69	VDAG_08233	256	1.6	CAS1 protein
	9.83	VDAG_04795	294	1.4	Cellulose-growth-specific protein
2.21		VDAG_02293	251	2.0	Chitin deacetylase
2.06	3.45	VDAG_00801	400	2.5	Cip2
3.12	4.85	VDAG_09287	235	3.4	FCY1p
3.19	2.02	VDAG_01694	336	2.7	Feruloyl esterase C
3.25	4.43	VDAG_09527	375	1.6	Hydrolase
8.37	10.12	VDAG_00261	205	2.0	IDI-3 protein
4.00	5.37	VDAG_00769	140	2.9	Infection structure specific protein
7.62	9.23	VDAG_10511	200	2.5	Infection structure specific protein
2.61	2.41	VDAG_07904	301	1.3	MAS1 protein
4.48	6.77	VDAG_01264	342	2.3	Mono
2.18	2.88	VDAG_02462	346	1.2	Nuclease S1
2.18	2.88	VDAG_09549	294	1.4	Oxidoreductase
7.01	8.44	VDAG_09774	355	2.0	Parallel beta-helix repeat protein
5.39	4.92	VDAG_07745	384	1.3	Peptide hydrolase
	3.02	VDAG_10378	292	4.1	Pheromone protein 1
3.35		VDAG_00559	179	2.2	Phosphatidylglycerol/phosphatidylinositol transfer protein
3.49		VDAG_01131	345	1.2	Proclavaminate amidinohydrolase
	2.37	VDAG_09550	396	1.3	Protein disulfide-isomerase
6.43		VDAG_09343	213	1.9	PRY1 protein
5.55	7.28	VDAG_01687	238	2.5	Rhamnogalacturonan acetylesterase
8.32	14.27	VDAG_05798	274	1.5	Rhamnogalacturonan acetylesterase
5.03	6.09	VDAG_05416	122	3.3	Ribonuclease T1
5.25	6.11	VDAG_07494	206	1.9	Secreted protein
7.81	10.94	VDAG_09117	277	1.8	Secreted protein
5.92	7.95	VDAG_09555	174	4.0	Secreted protein
9.04	12.18	VDAG_02908	211	2.8	Secretory phospholipase A2
13.83	13.21	VDAG_04019	187	2.1	Secretory phospholipase A2
3.71	7.98	VDAG_04026	261	1.5	Spherulin-1A
6.74		VDAG_01910	396	3.8	Thaumatin family protein
2.33	3.41	VDAG_04446	227	3.1	YoaJ
6.83	9.54	VDAG_00795	385	1.6	Zinc carboxypeptidase A 1
	8.29	VDAG_03707	321	2.8	Alpha-N-arabinofuranosidase
0.00	13.50	VDAG_07708	236	2.5	Cellulose-growth-specific protein
3.93	2.13	VDAG_02469	305	1.6	Glucan 1,3-beta-glucosidase
6.93	7.62	VDAG_05483	349	1.7	Lysophospholipase A
0.00	13.56	VDAG_02105	179	4.5	Uncharacterized protein
2.69	1.77	VDAG_02420	106	4.7	Uncharacterized protein
3.10	3.35	VDAG_02704	345	1.4	Uncharacterized protein
10.55	14.79	VDAG_02705	108	5.6	Uncharacterized protein
4.74	3.86	VDAG_03190	260	1.9	Uncharacterized protein
14.20	16.71	VDAG_03369	191	3.7	Uncharacterized protein
3.93	7.98	VDAG_03799	163	11.0	Uncharacterized protein
16.11	18.33	VDAG_03991	280	6.4	Uncharacterized protein
4.73	4.47	VDAG_04031	261	1.5	Uncharacterized protein
4.29	7.42	VDAG_04434	206	7.3	Uncharacterized protein
3.55	0.00	VDAG_04705	153	5.2	Uncharacterized protein
4.19	4.03	VDAG_04904	276	1.8	Uncharacterized protein
1.22	2.34	VDAG_05722	237	2.5	Uncharacterized protein
1.59	2.01	VDAG_06146	328	1.8	Uncharacterized protein
1.92	2.66	VDAG_06202	210	2.9	Uncharacterized protein
7.61	3.29	VDAG_06933	159	5.0	Uncharacterized protein
4.44	4.93	VDAG_06954	289	1.4	Uncharacterized protein
4.51	3.62	VDAG_08819	167	2.4	Uncharacterized protein
5.20	5.60	VDAG_09678	242	1.7	Uncharacterized protein
0.90	2.82	VDAG_09853	306	1.3	Uncharacterized protein

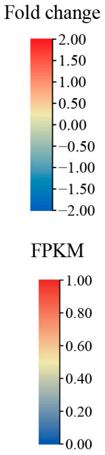

Figure S2. Heatmap of remaining up-regulated DEGs encoding SCRPs. The heatmap was generated based on fold change and FPKM values. Numbers in heatmap are fold change, with those greater than or equal to 2 displayed. Negative values indicate down-regulation of DEGs, and positive values indicate up-regulation of DEGs.

Table S1 Primer sequences used in this study

Gene name	Primer name	Primer sequence（5′–3′）
Tubulin	*Tubulin*-F	TTTCCAGATCACCCACTCC
	Tubulin-R	ACGACCGAGAAGGTAGCC
Ve-ITS1	*Ve-ITS1*-F	AAAGTTTTAATGGTTCGCTAAGA
ST-VE1	*ST-VE1*-R	CTTGGTCATTTAGAGGAAGTAA
GhUBQ7	*UBQ7*-F	GAAGGCATTCCACCTGACCAAC
	UBQ7-R	CTTGACCTTCTTCTTCTTGTGCTTG
pSUC2- *VdEP1* (SP)	pSUC2- *VdEP1* (SP)-F	GGAATTTTAATTAAGAATTCATGAAGACTCTCGCCCTCGTC
	pSUC2- *VdEP1* (SP)-R	ACTATAGGGAGAACCTCGAGCGTCAAGACACTCTGATCGATCC
PYBA-1132: *VdEP1*	PYBA-1132: *VdEP1 -F*	TAGAACTAGTGGATCATGAAGACTCTCGCCCTCGTC
	PYBA-1132: *VdEP1 -R*	CGGTATCGATAAGCTCGTCAAGACACTCTGATCGATCC
pSUC2- *VdEP1* (ΔSP)	pSUC2- *VdEP1* (ΔSP)-F	GGAATTTTAATTAAGAATTCAACACCATCTTCGTCAGCCAAA
	pSUC2- *VdEP1* (ΔSP)-R	ACTATAGGGAGAACCTCGAGCGTCAAGACACTCTGATCGATCC
pTRV2-*VdEP1*	pTRV2-*VdEP1*-F	AAGGTTACCGAATTATGAAGACTCTCGCCCTCGTC
	pTRV2-*VdEP1*-R	CTCGGTACCGGATCTGACGTTGAGCTGCTCGGT

Table S2 Primer list for gene validation by RNA-Seq analysis

Primer name	Primer sequence（5′–3′）
VDAG_07768 -qF	AAGAAGGGCCAGACCTTTGACG
VDAG_07768 -qR	GTTGGCGCCAATGATGACGTTA
VDAG_06165 -qF	CGCACTGTATGTAGCGATGGATCA
VDAG_06165 -qR	TTGCTCCAGCTCACGCTGTAC
VDAG_05708 -qF	ATCATCGGACCCAAAGTCAAGC
VDAG_05708 -qR	AGAAAATCAGCAAAAGTGCGTGG
VDAG_05640 -qF	GGCTCAATTTCCCCTATGTGCTG
VDAG_05640 -qR	GACAGCTATTTGGGTGGGAGAAGA
VDAG_03354 -qF	TTTCATCAAGTCAAACGCCGCC
VDAG_03354 -qR	CACTCGGCGGAGACAAGACTAA
VDAG_00808 -qF	CAGCGGAGATTGTTCCTCGAG
VDAG_00808 -qR	GCCATAAGTCTCAGAAAGACGAGC
Gohir.A02G136300 -qF	GATTACTGGTGACATAAACGTCTCC
Gohir.A02G136300 -qR	AAGTTCAGACATGAAGAGATTTCACG
Gohir.D12G265700 -qF	TAGTTATCGGTTCAAGTGGGTTCA
Gohir.D12G265700 -qR	ATTCTTGGTCGGTGATAGAACCAT
Gohir.D03G047100 -qF	TGCTTCAAAGCTGGGTACCTAA
Gohir.D03G047100 -qR	TGTTGGCTTTCAACAAAGTAAGGG
Gohir.A12G002900 -qF	GTTGGCAATGTCTTCATCTCCG
Gohir.A12G002900 -qR	TCAGTAGTAAGACGGGGAATCTTT
Gohir.D05G088200 -qF	TTCCTACATCAATGATCTAAAGGCGA
Gohir.D05G088200 -qR	CCAGTTCCAGGAGATGAATAGCT
Gohir.D10G144300 -qF	TTGCTTCGCAGGGGGGA
Gohir.D10G144300 -qR	ATAACAGCTGCGGCACCATC
